# Supplementary material for: Complexome profiling of the Chlamydomonas psb28 mutant reveals TEF5 as an early PSII assembly factor
Source: Plant Cell. 2025 Mar 17;37(6):koaf055. doi: 10.1093/plcell/koaf055 (PMC12164590; doi:10.1093/plcell/koaf055)
Supplement: koaf055_Supplementary_Data [file koaf055_supplementary_data.zip › psb28_interactive.zip!/plots/intensity/qualityPlots/peptides/LHCBM8_Cre06.g284250.t1.2.html]

### Cre06.g284250.t1.2

Trivial name: LHCBM8  
SeqCoverage [median]: 61.00  
Mass: 27.03 kDa  
  
 

| Sequence | MissedCleavage | Mass [Da] | wtIntensity | muIntensity | Score |
| --- | --- | --- | --- | --- | --- |
| GPVQNLDDHLANPGVNNAFAFATK | 0 | 2509.24 | 173012.75 | 82721.24 | 229.35 |
|  |  |  | 173012.8 | 82721.2 |  |
